# Pushing the Limits: The Pattern and Dynamics of Rubber Monoculture Expansion in Xishuangbanna, SW China

**DOI:** 10.1371/journal.pone.0150062

**Published:** 2016-02-23

**Authors:** Huafang Chen, Zhuang-Fang Yi, Dietrich Schmidt-Vogt, Antje Ahrends, Philip Beckschäfer, Christoph Kleinn, Sailesh Ranjitkar, Jianchu Xu

**Affiliations:** 1 Key laboratory of Biodiversity and Biogeography, Kunming Institute of Botany, Chinese Academy of Sciences, Kunming, China; 2 World Agroforestry Centre (ICRAF) East and Central Asia, Kunming, China; 3 Mountain Societies Research Institute, University of Central Asia, Bishkek, Kyrgyz Republic; 4 Royal Botanic Garden Edinburgh, Edinburgh, United Kingdom; 5 Chair of Forest Inventory and Remote Sensing, Georg-August-Universität Göttingen, Göttingen, Germany; Lakehead University, CANADA

## Abstract

The rapidly growing car industry in China has led to an equally rapid expansion of monoculture rubber in many regions of South East Asia. Xishuangbanna, the second largest rubber planting area in China, located in the Indo-Burma biodiversity hotspot, supplies about 37% of the domestic natural rubber production. There, high income possibilities from rubber drive a dramatic expansion of monoculture plantations which poses a threat to natural forests. For the first time we mapped rubber plantations in and outside protected areas and their net present value for the years 1988, 2002 (Landsat, 30 m resolution) and 2010 (RapidEye, 5 m resolution). The purpose of our study was to better understand the pattern and dynamics of the expansion of rubber plantations in Xishuangbanna, as well as its economic prospects and conservation impacts. We found that 1) the area of rubber plantations was 4.5% of the total area of Xishuangbanna in 1988, 9.9% in 2002, and 22.2% in 2010; 2) rubber monoculture expanded to higher elevations and onto steeper slopes between 1988 and 2010; 3) the proportion of rubber plantations with medium economic potential dropped from 57% between 1988 and 2002 to 47% in 2010, while the proportion of plantations with lower economic potential had increased from 30% to 40%; and 4) nearly 10% of the total area of nature reserves within Xishuangbanna has been converted to rubber monoculture by 2010. On the basis of our findings, we conclude that the rapid expansion of rubber plantations into higher elevations, steeper terrain, and into nature reserves (where most of the remaining forests of Xishuangbanna are located) poses a serious threat to biodiversity and environmental services while not producing the expected economic returns. Therefore, it is essential that local governments develop long-term land use strategies for balancing economic benefits with environmental sustainability, as well as for assisting farmers with the selection of land suitable for rubber production.

## Introduction

Since the 20th century, rubber tree (*Hevea brasiliensis)* plantations have been expanding rapidly throughout Southeast Asia [1–3], which currently supplies over 90% of the world’s natural rubber [4]. While rubber tree planting in Southeast Asia prior to the 1990s was mainly confined to southern Thailand, Malaysia, and Indonesia, the focus of plantation establishment has recently shifted north, where since the 1990s, a rubber boom, mainly driven by China’s emerging car industry, has led to a rapid expansion of monoculture rubber plantations in the Mekong region. More than one million hectares of land have been converted into rubber plantations in Laos, Thailand, Vietnam, Cambodia, Myanmar, and South China [5].

China’s natural rubber consumption greatly outstrips its production. For instance, in 2010, China consumed 33% of the global natural rubber production [6] while only producing about 7% [4]. In order to close this gap and to increase its domestic supply with natural rubber, China has implemented a range of measures, such as subsidizing smallholder rubber farmers, establishing state rubber farms, developing cold-and dry-resistant tree varieties, and providing training for rubber farmers.

From among the three rubber producing provinces in China (Hainan, Yunnan, and Guangdong), Yunnan Province ranks second in terms of natural rubber production. Xishuangbanna Prefecture, located in Yunnan’s tropical south, accounts for 77% of the rubber produced in the province, and for 37% of the national production [7, 8]. Since the 1980s, the rapid growth of China’s rubber production is largely due to the expansion of rubber plantations in Xishuangbanna. Rubber plantations in Xishuangbanna Prefecture were originally established in the 1950s in eight state farms at elevations below 900 meters above sea level (masl.). This was in response to Chinese military needs during the Korean War, which elevated natural rubber to the status of a strategic, industrial product [9].

With rubber prices having tripled over the last decade, the production of rubber has become an extremely lucrative business for both the public sector and for farmers in Xishuangbanna. In 2008, rubber contributed to up to one third of local government revenues, and to roughly half of the household income of farmers in Xishuangbanna [10]. Rubber production is, therefore, heavily promoted by the government and has become a priority measure for poverty alleviation, especially for minority ethnic groups in Xishuangbanna Prefecture and Hainan Province.

Xishuangbanna is located within the Indo-Burma biodiversity hotspot, and supports a very high diversity of flora and fauna. In line with the 2011–2030 China Biodiversity Conservation Strategy and Action Plan, Xishuangbanna has been declared a territorial biodiversity conservation priority by the Ministry of Environmental Protection of China [11]. The prefecture’s biodiversity is, however, threatened by a decrease of natural forest cover from around 70% in the 1970s to 50% in the 2000s due to the expansion of rubber plantations [12–16].

Rubber plantations have since recently been expanding into areas that are unsuitable for rubber growing due to climatic and topographic conditions [17–19]. Most of the rubber plantations above 900 masl. were established by smallholders and private investors who may have had insufficient knowledge of the hazards and risks of growing rubber at high altitudes [20–22]. Rubber plantations in such marginal growing environments come with low production. Smallholder rubber plantations have increased their share of the total rubber plantation area from 54% in 2003 to 62% in 2008 [23].

In order to avoid or mitigate the negative effects of rubber plantation expansion, land use planning should ideally be based on a good understanding of the spatial extent, pattern, and dynamics of rubber plantations and their economic potential, and how they affect forest cover and biodiversity. Transparent and spatially explicit information is a prerequisite for such an understanding.

Remote sensing offers the possibility to produce spatially explicit information to support land use planning, and various mapping studies for rubber plantations were done over the years. However, most remote sensing based mapping of rubber plantations in Southeast Asia was done from low and medium spatial resolution imagery. Li and Fox [5] used Moderate Resolution Imaging Spectroradiometer (MODIS) imagery from 2009 and 2010 to map rubber plantations in mainland Southeast Asia and Landsat TM imagery from 2004 and 2009 to map different plantation ages in Northeast Thailand [24]. Dong et al. [25] mapped rubber plantations and forests in 2009 on Hainan Island with a combination of Phased Array type L-band Synthetic Aperture Radar (PALSAR) images and Landsat TM images. In 2005, Advanced Spaceborne Thermal Emission and Reflection Radiometer (ASTER) imagery was applied for rubber plantation mapping at the Thai-Lao and Sino-Lao borders [26]. Hurni [27] adopted ASTER imagery from 2001 and 2006 to map the dynamics of rubber plantations in Laos. In Malaysia, the area, volume, and age of rubber plantations was estimated using Landsat TM imagery [28]. Zhai et al. [29] used Landsat and SPOT images to detect changes of rubber plantations and pulp plantations between 1988, 1995, and 2005 in Changhua watershed on Hainan Island, South China. Liu et al. [18] used Landsat MSS/TM/ETM and MODIS imagery to detect expansion of rubber plantations between 1980 and 2010 in the border region of China, Laos, and Myanmar, which included the Xishuangbanna Prefecture. It was found that the area of rubber plantations expanded in this region by six times, from 70,500 ha in 1980 to 501,000 ha in 2010 [18].

In Xishuangbanna, Landsat MSS, Landsat TM, and Landsat ETM images were used for mapping land cover in 1976, 1988, and 2003, respectively [12]. It was found that rubber plantations had increased from 21,065 ha or 1.1% of the total land area in 1976 to 216,395 ha or 11.3% in 2003. Senf et al. [30] applied phenological metrics derived from MODIS for mapping of rubber plantations and forests in Xishuangbanna Prefecture in 2010, with an overall accuracy of 74%. According to their assessment, rubber plantations covered 30% (±4%), which suggests a tripling of the area under rubber when compared with the figure of Li et al. [12] from 2003.

Although some analysis of rubber expansion in relation to topographic factors (e.g. elevation and slope) has been conducted for Xishuangbanna Prefecture [12, 13, 18], the spatial distribution of economic benefit of rubber plantations in the area and the extent of rubber plantation encroachment into areas of high conservation value have not yet been determined. Our paper tries to fill this gap.

Moreover, this is the first time that high-resolution imagery (RapidEye, 5 m resolution) is applied to mapping rubber in this area. We used high-resolution imagery that included RapidEye, which has a red-edge spectral band. Combining red-edge spectral band with other spectral bands allows for better identification of vegetation, and, in the case of our study, enabled us to distinguish between young (open canopy) and mature (closed canopy) rubber plantations.

In this study, our main objectives were: 1) to do an accurate and consistent assessment of the spatial distribution and changes over time of rubber plantations in Xishuangbanna, 2) to determine the extent and rate of rubber expansion into areas of high biodiversity and conservation value, and 3) to conduct a spatially explicit assessment of the economic value of rubber plantations described by their net present value (NPV). These objectives are based on the hypothesis that expansion of rubber plantations into higher elevations where most of the remaining forests are located and which are unsuitable for rubber cultivation, poses a threat to biodiversity, while not producing the expected economic returns. As the recent expansion of rubber monocultures is largely driven by smallholders, the results of this study are expected to provide a more solid basis for planning and extension services. Local governments and research institutes can use the results presented in this paper as a reference point for future research on the drivers and effects of rubber plantation expansion, as well as for initiatives to limit expansion of rubber plantations into unsuitable areas, and to promote sustainable and environmentally-friendly rubber cultivation such as intercropping rubber with timber trees and medicinal plants.

## Materials and Methods

### Study area

**Fig 1:**
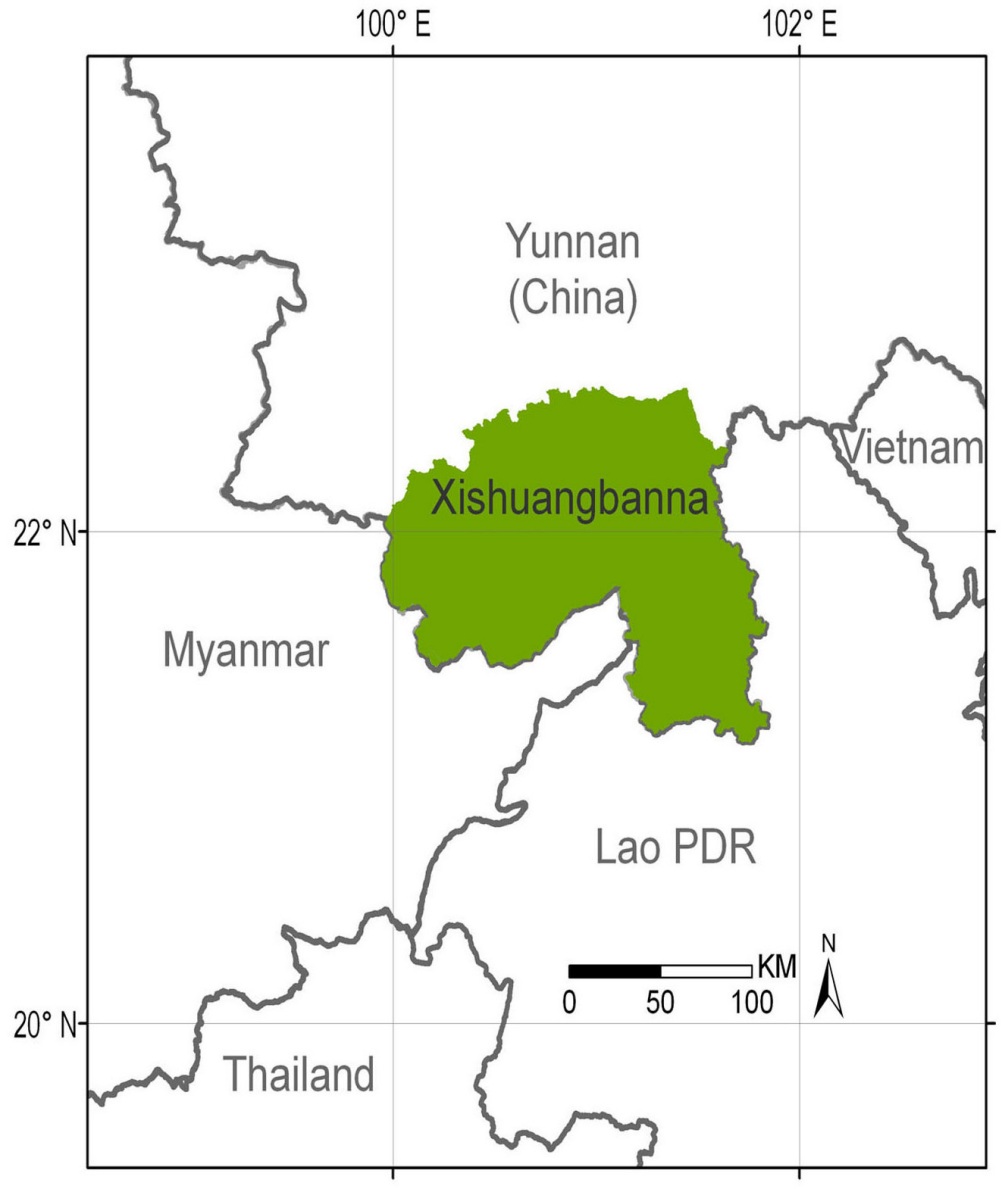
Location of Xishuangbanna Prefecture in the Mekong region.

The Xishuangbanna Dai Autonomous Prefecture is located in southern Yunnan, China, bordering Laos PDR and Myanmar (Fig 1). With elevations ranging from 475 to 2,428 masl., most of the total prefecture area (19,164 km^2^) is categorized as mountainous [13]. Xishuangbanna has a tropical and sub-tropical monsoon climate, with a wet season from May to October, which provides about 85% of the annual rainfall. The average annual temperature is around 22°C, and the average annual rainfall is 1,317 mm [31]. Five types of primary forest occur in Xishuangbanna: tropical seasonal rainforest (< 900 masl.), tropical montane rainforest (700–1,500 masl.), monsoon forest situated on riverbanks (< 900 masl.), monsoon forest located on limestone (< 800 masl.), and sub-tropical evergreen broad-leaved forest (1,000–1,500 masl.) [32].

Xishuangbanna is also culturally diverse. In 2010, 78% of its population of 1.1 million people belonged to ethnic minorities, including the valley-dwelling Dai and upland ethnic groups such as Hani, Jinuo, Yao, Lahu, and Bulang [33].

#### Satellite remote sensing for rubber plantation mapping

Rubber plantations were mapped based on a Landsat TM image and a Landsat ETM+ image (path/row number: 130/45, acquisition dates: 2 Feb. 1988 and 28 Mar. 2002, spatial resolution of 30 m). Radiometric and geometric rectification has been applied for these images (L1T products) by the imagery supplier. For 2010, a recently published map of rubber distribution in Xishuangbanna [34] and the corresponding RapidEye satellite images were re-analyzed using a refined classification scheme which allows for distinguishing open canopy rubber plantations from closed canopy rubber plantations. The 48 RapidEye (RE, ortho product level 3A, spatial resolution of 5 m) scenes were captured during January and February of 2010. The TM, ETM+, and RE images were registered in Universal Transverse Mercator Projection (UTM WGS 84).

A land cover classification scheme was developed, distinguishing open and closed canopy rubber plantations, forests, agricultural land, grass/shrub land, water, as well as built-up and bare land. Closed canopy and open canopy rubber plantations represent mature (> six years old) and young (< six years old) plantations, respectively. This distinction has been quantified empirically by verification with differently aged rubber tree plantations on Google Earth Quickbird imagery. We obtained 460 GPS points of rubber plantations in the study area during the period 2009 until 2011. For 308 out of these 460 points, tree age information was recorded. We determined a crown coverage of 45% as threshold value that separates young plantations (< 45% crown cover) from mature plantations (> 45%) by selecting points of rubber trees younger than six years in the field, then overlaying on high-resolution images on Google Earth Quickbird imagery, and calculating crown coverage. Image classification was done with the software eCognition 8.0 (Trimble, U.S.) using membership function and nearest neighbor classifiers. Associated threshold values of certain object features were determined and implemented in class membership functions. The object-based classification using the membership function classifier was also applied for identification of rubber plantations in Landsat images.

Training areas were acquired from ground truth GPS points and high-resolution satellite images accessible on Google Earth (DigitalGlobe, U.S.) [35]. RE images were classified scene by scene and subsequently merged.

Freely accessible DigitalGlobe archives played an important role in accuracy assessment of classification [36]. Reference points for accuracy assessment were created randomly. 71 points of rubber plantation were generated based on high-resolution images from Google Earth from 2001 and 2002 as references to verify the identification of rubber plantations for 2002. For verification of RE image classification for the year 2010, a combination of 361 points were used from the field in 2009/2010 and from Google Earth at around the same time.

### Spatial data preparation

Administrative data including boundaries of Xishuangbanna Prefecture, its counties, boundaries of nature reserves, as well as a digital elevation model (DEM), scale 1:50,000, 25 m resolution, were obtained from the Centre for Mountain Ecosystem Studies (Kunming Institute of Botany, Chinese Academy of Sciences, China). A 100 m-interval elevation layer, as well as layers of slope and aspect, were derived from the DEM. The DEM layer was resampled from 25 m to 30 m horizontal resolution, and the layer of rubber plantations in 2010 derived from RE images was also resampled to 30 m to match the resolution with those of 1988 and 2002. As it turned out, resampling of the layer of rubber plantations in 2010 from 5 m to 30 m resulted in a change of less than 1% of the total rubber plantation area.

For analyzing productivity and the economy of rubber plantations, we assessed the economic value of rubber plantations in different locations by using a map (30 m resolution) produced by Yi et al. [20, 37] depicting the net present value (NPV) of rubber plantations in their average lifespan of 25 years. NPV signifies the difference between capital and resource investments and the benefits over a specific time period, as a function of rubber productivity, average rubber price at the local market, costs of plantation establishment and management, length of plantation rotation period, and the social discount rate (for a detailed description see [20, 37]) from:

$$NPV=\frac{\sum_{0}^{25}\left(B_{i}-C_{i}\right)}{{\left(1+r\right)}^{i}}$$ (1)

where *i* = 0, 1, 2…25 (25 years is the average rotation length of rubber plantations in the study area); *B*_*i*_ is the expected benefit of a rubber plantation in the *i*th year of the rotation period; and *C*_*i*_ represents the establishment and management costs in the *i*th year. Rubber productivity, which is one input to calculate *B*_*i*_, was modeled using a multivariate linear regression of rubber productivity data collected in the field (*n* = 468) against environmental predictors such as elevation, slope, rainfall, soil features, and temperature. A social discount rate of 8% for China was used in this study as suggested by the World Bank [20, 38], and a local average price of 3,400 US$ ton^−1^ [20, 37]. NPV as a locally specific value indicates whether rubber trees at specific locations eventually become profitable or not, and what is the value of economic benefits that rubber trees can provide in their average lifespan.

All maps were projected to a common grid system, the Universal Transverse Mercator (UTM) WGS84 reference system so as to conduct overlay analysis; all map spatial analysis was done in ArcGIS 9.3 (ESRI Inc., U.S.).

### Analysis

Raster layers of rubber plantations in 1988, 2002, and 2010 were overlaid with elevation and slope layers, administrative boundaries, and the boundaries of nature reserves. Mean elevation and slopeof rubber plantations were calculated for each of the three years and compared in order to assess plantation shifts in elevation and slope.

We applied two-tailed t-tests (allowing for unequal variances) to test whether mean elevation and slope of plantations had changed significantly over the study period 1988–2010. These statistical analyses took place in R 3.0.1 [39]. An overlay analysis of NPV and land cover layers from the three years was conducted for a spatially explicit assessment of the economic value of rubber plantations.

## Results

### Accuracy assessment

The accuracy of rubber plantation identification for 2002 is 88.7% and the overall accuracy for all land cover classes for 2010 is 87.5%, with a Kappa coefficient of 0.82.

### Geographic distribution and expansion

**Fig 2:**
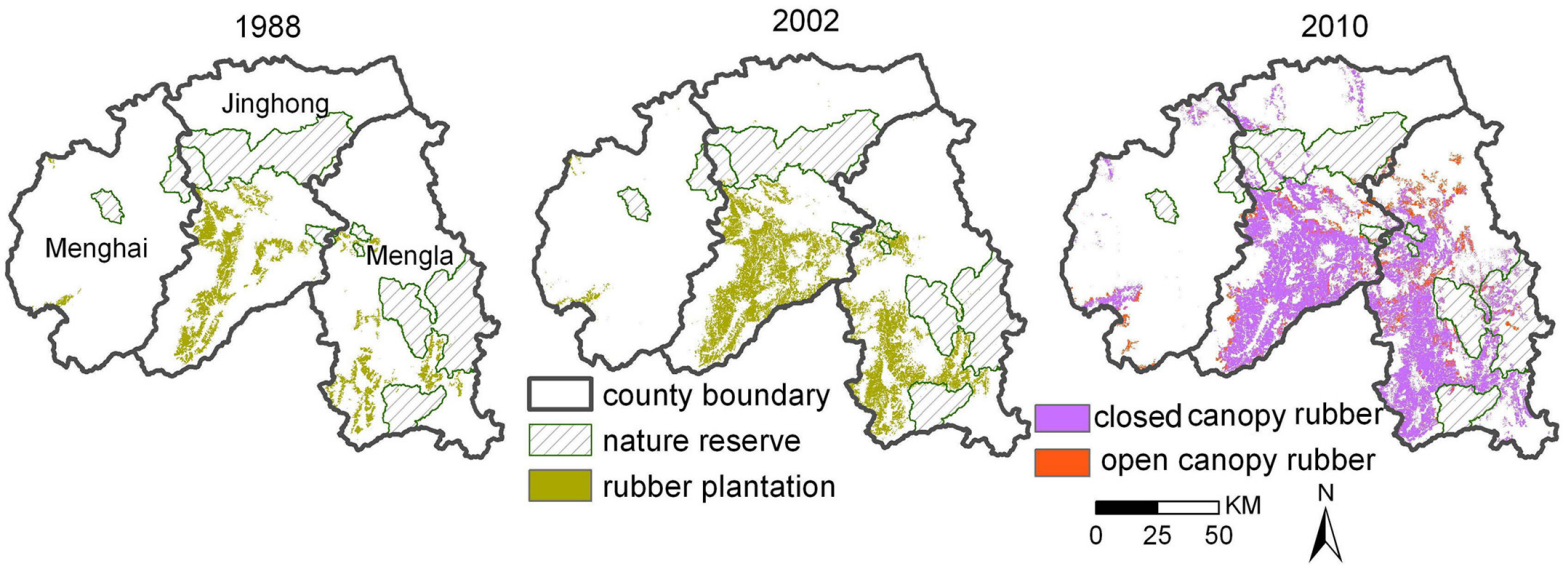
Rubber plantations in 1988, 2002, and 2010.

Rubber plantations were mapped for the years 1988, 2002, and 2010 (Fig 2), with the accuracy of 88.7% for 2002 Landsat images and 90.0% for 2010 RE images (87.5% overall accuracy for all land use classes). The maps (Fig 2) indicate that, during this period, the area of rubber plantations nearly quintupled from 87,111 ha in 1988, to 189,764 ha in 2002, and to 424,552 ha in 2010 (compare Xu et al. 2014 [34]). In 2010, rubber plantations were composed of 88.6% closed canopy rubber plantations and 11.4% open canopy plantations.

Between 1988 and 2010 the area utilized for rubber cultivation in Xishuangbanna has increased on average by 15,338 ha yr^−1^. The greatest increase took place between 2002 and 2010 when approximately 55.3% of the currently planted area came under rubber. The annual expansion of rubber plantations increased from 7,332 ha yr^−1^ between 1988 and 2002 to 29,349 ha yr^−1^ between 2002 and 2010. The proportion of land covered by plantations increased from 4.5% in 1988, to 9.9% in 2002, to 22.2% in 2010.

Comprising more than 90% of the rubber plantation area of Xishuangbanna, the two counties of Jinghong and Mengla are the main areas for rubber growing and plantation expansion (Fig 2). This is mainly due to the large proportion of land at low elevations that offers favorable conditions for rubber tree plantations with 88% of the land being located below 900 masl. Another important factor is that cultivation of rubber in Xishuangbanna was initiated in the 1950s in eight state farms in the lowlands of Jinghong and Mengla County and later adopted by smallholder farmers as an economically-convincing land use and production system.

**Fig 3:**
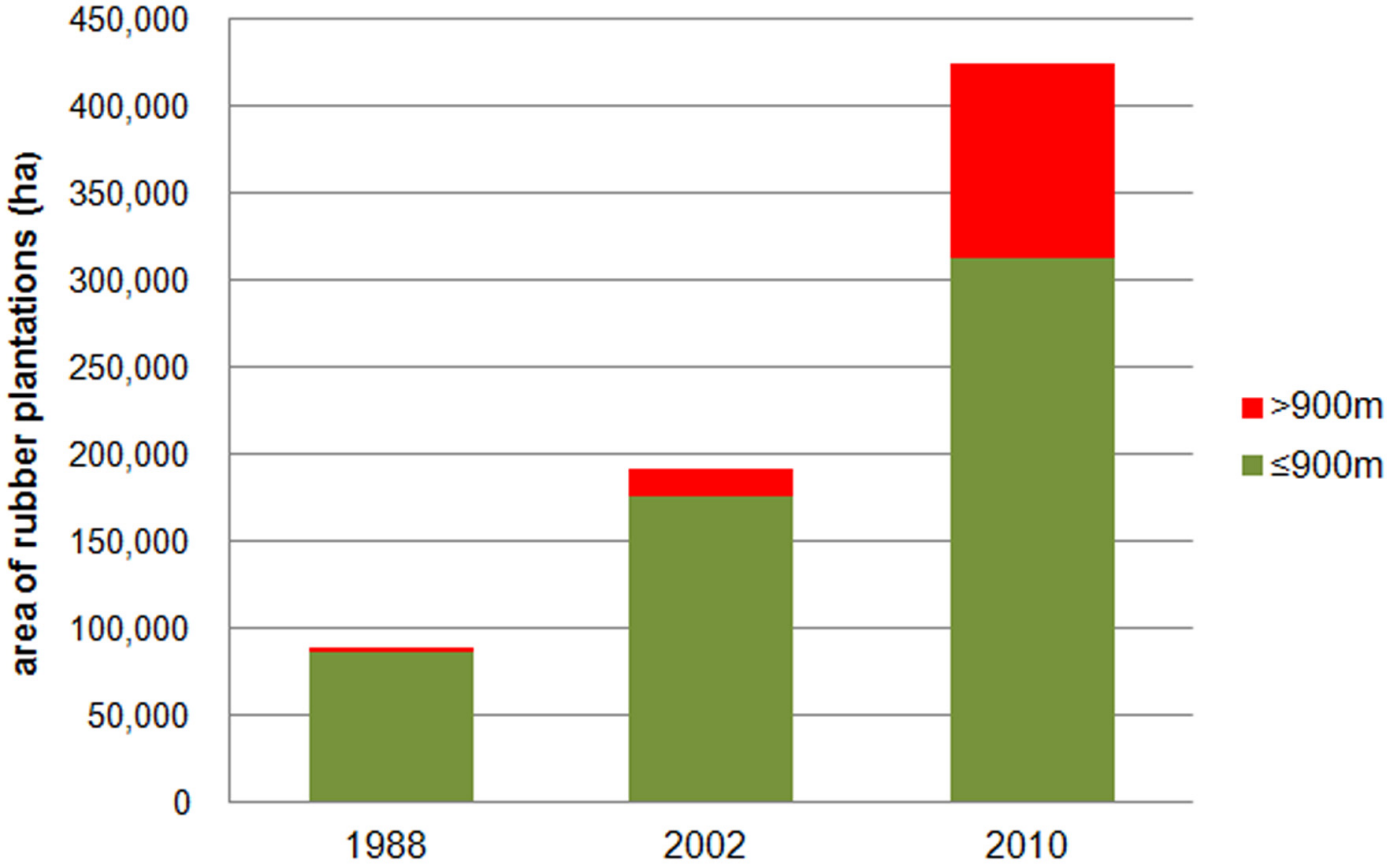
Expansion of rubber plantations in elevation zones.

**Fig 4:**
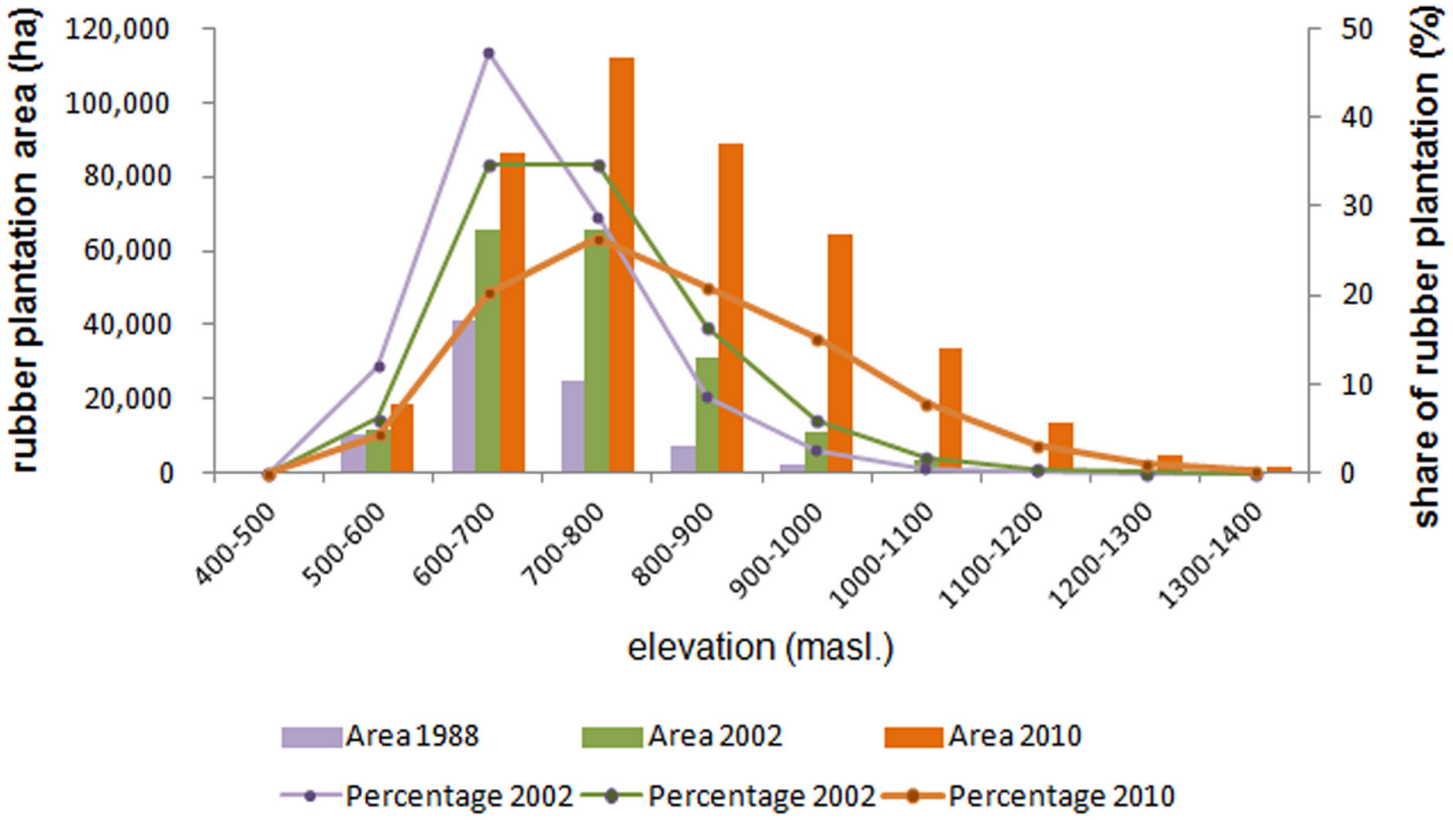
Distribution of rubber plantations (area and percentage) on elevation categories in 1988, 2002, and 2010.

While most rubber expansion occurred below 900 masl. (84,236 ha in 1988 to 305,673 ha in 2010), a noticeable increase of area under rubber plantations also took place at elevations above 900 masl., where rubber plantation area has increased forty times (2,875 ha in 1988 to 118,879 ha in 2010) (Fig 3). The upper limit of rubber plantations was 1,100 masl. in 1988, 1,300 masl. in 2002, and 1,400 masl. in 2010, and their mean elevation was 693 masl., 732 masl., and 815 masl., respectively. Despite this trend of upward expansion, the majority of rubber plantations was located at elevations below 900 masl. throughout the study period 1988–2010. Consequently, the proportion of plantations below 900 masl. has gradually diminished from 97.1% in 1988, to 93% in 2002, and to 73.7% in 2010 (Fig 4).

In 2010, mature (closed canopy) plantations had a mean elevation of 808 masl. (±156 SD) compared to a mean elevation of 958 masl. (±145 SD) of young (open canopy) plantations; a difference which is highly significant (t = -826.5372, p ≤ 0.001). This indicates that rubber expansion is a process of new plantations being established at higher elevations (i.e. in low profit rubber cultivation areas, potentially without economic returns in the long-run).

While the area of rubber plantations has expanded in all elevation categories where rubber trees are capable of growing, the pace of expansion was more rapid at higher altitudes. From 1988 to 2002, rubber plantations have expanded most rapidly at an elevation of 700–800 masl., where 39.6% of the new rubber plantations (40,609 ha) were established. From 2002 to 2010, the zone of maximum rubber expansion with 47.5% of new rubber plantations shifted to elevations of 800–1,000 masl. In total, about 50% of new rubber plantations were established in elevations between 700–900 masl. in the 22 years from 1988 to 2010.

**Fig 5:**
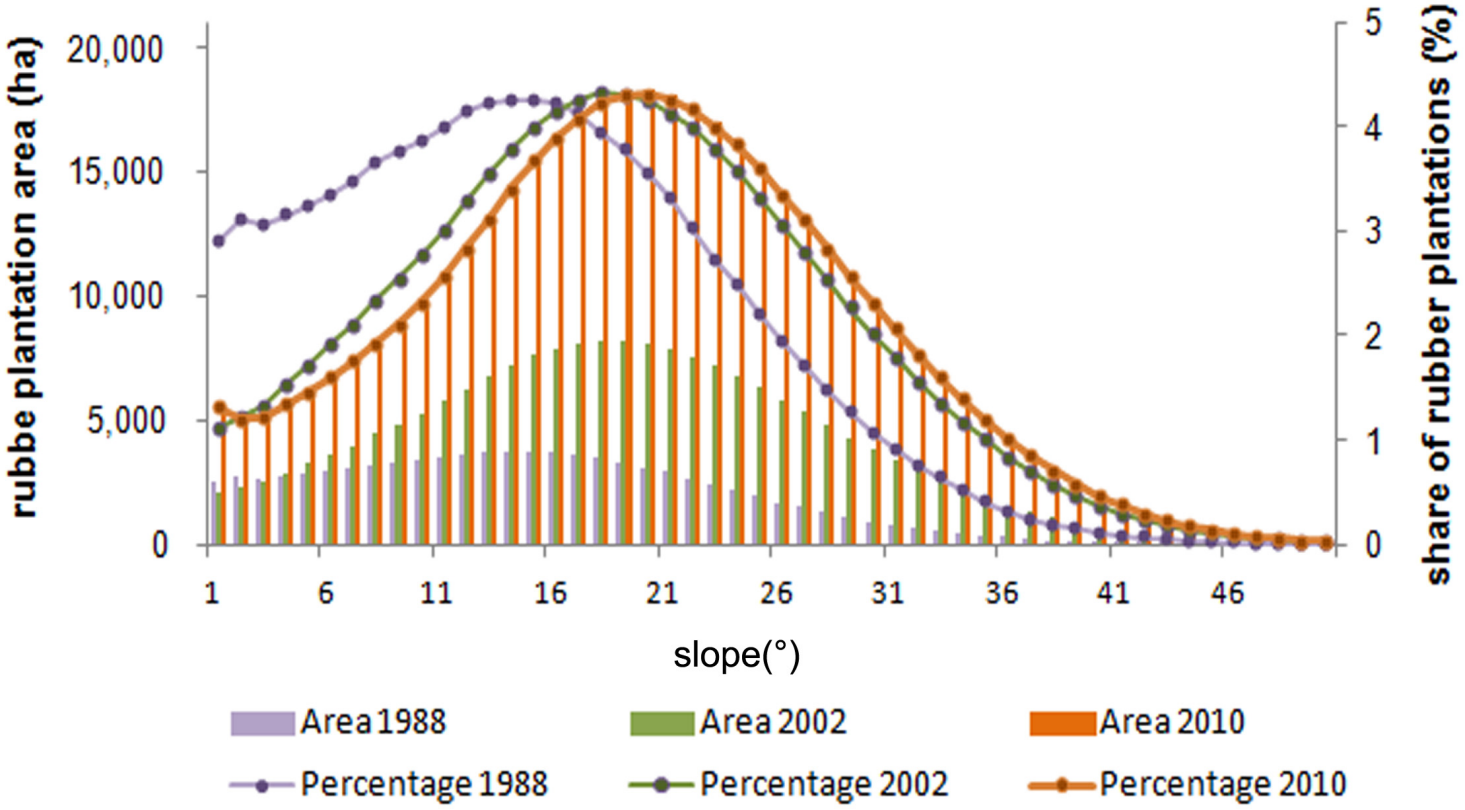
Distribution of rubber plantations in area and percentage on slope categories in 1988, 2002, and 2010.

Xishuangbanna Prefecture is characterized by rugged terrain, with slopes steeper than 15 degrees accounting for 71% of the total land area. Fig 5 shows the distribution of rubber plantation area over slope categories for 1988, 2002, and 2010. There is a clear trend towards plantations established on steeper slopes. The mean slope of rubber plantations has increased significantly from 14.6° (±7.8 SD) in 1988, to 17.9° (±8.7 SD) in 2010 (t = -373.27, p ≤ 0.001). In 2010, mature plantations had a mean slope angle of 17.6° (±8.7 SD) and young plantations a mean slope of 19.9° (±8.8 SD). This difference was highly significant (t = -207.7941, p ≤ 0.001). The largest proportion of rubber plantations was on slopes of 15° in 1988, on slopes of 18° in 2002, and on slopes of 20° in 2010. Conversely, the proportion of rubber plantations on relatively flat terrain with slopes < 15°, has dropped from 54.5% in 1988, to 36.2% in 2002, and to 31.9% in 2010. Between 1988 and 2010, 49.8% of rubber plantation area increases occurred on slopes of 15°-26°. With respect to the slope aspect, we found no recognizable trend in Xishuangbanna of rubber plantation establishment.

The total area of forest excluding tree plantations in Xishuangbanna was 1,065,843 ha (55.6% of the total land area) in 2010 with only 13% of forests growing in the lowlands below 900 masl., while 41.7% of forests in the uplands (i.e. above 900 masl.) are located in the altitudinal zone of 900–1200 masl., where rubber plantations have been expanding at a rapid pace. In 2010, 30.7% of this elevation range was covered by rubber plantations. Continued expansion of rubber plantations at this elevation would pose a particular threat to existing forests and their biodiversity

### Rubber plantations in nature reserves

Two national nature reserves were established in Xishuangbanna in 1959 and 1993, covering a total area of 241,776 ha (13% of the prefecture). The total area of rubber plantations in nature reserves has increased from 264 ha in 1988, to 2,113 ha in 2002, and 23,616 ha in 2010. In other words, since 1988 there has been an 89-fold expansion of the area under rubber cultivation in nature reserves. In 2010, rubber plantations made up 9.8% of the total nature reserves’ area, compared to 0.1% in 1988 and 0.9% in 2002.

### Rubber expansion and NPV

NPV represents the potential economic benefit from rubber plantations. In the time period between 1988 and 2010, the mean NPV of rubber plantations decreased by 8%, from 24,004 US$ ha^−1^ in 1988, to 22,044 US$ ha^−1^ in 2002, and to 19,088 US$ ha^−1^ in 2010. This trend runs parallel to the expansion of rubber plantations into higher elevations.

The overlay analysis of the NPV map with the rubber plantation map indicates that the frequency distribution of rubber plantations in the different NPV categories has not changed much between 1988 and 2002. During that time period, around 57% of rubber plantations had a medium level potential economic benefit of 20,000–40,000 US$ ha^−1^ (Table 1). However, in 2010, after rubber plantations had expanded to less productive land at higher elevations and on steeper slopes, the percentage of plantations with positive NPV declined, while the proportion of those having a negative NPV has increased. Around 10% of the newly established (that is: open canopy) plantations (2,179 ha) were found to bring no economic benefit at all, and 49.5% of them (23,958 ha) have only low net present values (< 20,000 US$ / ha^−1^).

**Table 1:** Proportions (%) of rubber plantation on rubber net present value (NPV).

NPV (US$ / ha^−1^)	1988	2002	2010	Closed canopy rubber (2010)	Open canopy rubber (2010)
< 0	3.7	3.3	8.4	8.1	10.7
0–20,000	30.4	34.1	39.6	38.4	49.5
20,000–40,000	57.4	57.8	46.9	48.7	32.5
40,000–63,080	8.5	4.8	5.1	4.8	7.3
Sub-total	100	100	100	100	100

## Discussion

The expansion of rubber plantations has been the primary factor for landscape transformation in Xishuangbanna in the past several decades [12, 15, 18, 21]. Between 1988 and 2010 their area has expanded from 4.5% to 22.2%. In the same time period, the price of natural rubber latex increased rapidly, from 130 US$ ton^−1^ in 2000 to 6,300 US$ ton^−1^ in 2011 [20, 40], presenting a strong incentive for smallholders to switch from previous land uses to lucrative rubber cultivation. In addition, government agencies promote rubber planting as a measure to economic growth in the prefecture, to enhance rural livelihoods and combat rural poverty.

While the vast majority of plantations was initially located below 900 masl., (i.e. at elevations known to be most suitable for rubber tree growth [41]), between 1988 and 2010 more and more rubber plantations were established at higher elevations where rubber growth is possible, but somewhat less productive so that by 2010 almost 30% of the plantations were located above 900 masl. Rubber plantations have also moved to steeper slopes with two thirds of the plantations now being on slopes of more than 15° inclination. Rubber plantations do currently occupy almost one quarter of the landscape, so that areas suitable for rubber plantations are becoming scarce. Liu et al. [18] in their study of the border region of China, Myanmar, and Laos confirm these trends and argue that the expansion of rubber plantations has reached its limits in China and is now likely to cross the border into Laos and Myanmar.

A study conducted by Yi et al. [20] has shown that rubber plantations located at elevations above 900 m or on slopes with an inclination of 24° or more are economically not profitable Rao et al. [42] have shown that optimum yields require minimum temperatures of 22.8°C. More importantly, rubber trees in marginal environments may be more prone to mortality due to greater climatic extremes [43]. According to our results, 60% of open canopy rubber plantations in 2010 had either negative, or very low NPVs. This is related to the fact that open canopy rubber plantations are located at higher elevations. Investing into these “low profit plantations” generates a lose-lose situation, where ecosystem services and biodiversity are lost and long-term financial gains are not achieved [20]. It must be considered a limitation of our study that we have been able to make the distinction between open and closed canopy rubber plantations only for the year 2010, and not for 1988 and 2002.

Previous studies have provided evidence that conversion to rubber plantations has been the cause of a loss of biodiversity in Xishuangbanna [44, 45]. This holds true especially for Jinghong County and Mengla County, which are particularly rich in biodiversity [46], and which are at the same time the main areas for rubber plantation expansion.

Expansion of rubber plantations affects biodiversity conservation, especially when occurring within protected areas. The forest type that is most seriously affected by rubber expansion is tropical seasonal rainforest, located below 900 masl., where most of the expansion of rubber plantations has taken place between 1988 and 2002. A total area of 139,576 ha, which corresponds roughly to two thirds of the area initially covered by this forest type, has been converted to rubber plantations between 1976 and 2003 [12]. However, most of the remaining forest cover of Xishuangbanna is located above 900 masl. This means that the expansion of rubber plantations into elevations between 900 and 1200 masl. that has been taking place since 2002 poses a serious threat of deforestation at higher elevations. It is already evident that the remaining forests are becoming increasingly fragmented [34, 47].

The conversion of tropical primary and secondary forests to rubber plantations has also been documented in Hainan Province, the largest natural rubber supplier in South China [29]. With deforestation advancing quickly, protected areas are the last refuges for threatened species [48]. However, our results clearly indicate that rubber plantations are also often established in nature reserves in China, with plantations covering almost 10% of the total nature reserve area of Xishuangbanna in 2010.

The results of our research confirm the hypothesis stated at the outset that rapid expansion of rubber plantations into higher elevations poses a threat to biodiversity while not providing the expected economic benefits. We are aware, however, that a limitation of our study is its restriction to analysis based on remote sensing and economic data without taking into account the perspective of smallholder farmers who are actually driving these changes. However, to carry out a participatory survey of farmers’ perceptions on a scale commensurate with the scale of our spatial analysis was beyond the scope of our resources and intentions.

Thus far, local farmers have benefited from rubber plantations. A survey conducted by the East and Central Asia (ECA) Regional Office of the World Agroforestry Centre, China, of 1,000 households in 50 villages within seven rubber growing townships has shown that rubber has become a major income source for smallholders’ households [49]. For instance, rubber plantations contribute up to 90% of household incomes in Menglun Township. However, the large-scale conversion of land (often forest) to monoculture rubber and the increasing proportion of plantations established in marginally-suitable areas may have long-term negative consequences for ecosystem services and sustainable household livelihoods [20]. The risk to livelihoods, largely based on the income from one single commercial crop has been illustrated dramatically by the recent drop in rubber prices. Currently, ongoing research by ECA in Manlin village of Xianming township and Mankong village of Menghan township has produced evidence that the response of farmers in Xishuangbanna to declining rubber prices is variable, ranging from maintaining plantations in the hope of price recovery to cutting down rubber plantations and replacing them with banana groves (Mertens, pers. comm). A similar pattern of rubber plantations being replaced by banana plantations as a response to falling prices has been reported from Luang Namtha across the border in Lao PDR, one of the main rubber producing areas in Lao PDR at the height of the rubber boom [50,51]. However, while the rubber trees cut down in Luang Namtha were between 8 and 10 years old (i.e. they had not entered the prime latex producing years), the rubber trees cut down in Xishuangbanna tended to be more mature trees [51]. Though it is too early for a conclusive statement on future trends of rubber prices, there is a possibility that continuing low prices may affect the dynamics presented in this paper and lead to a slowing down or even reversal of the expansion of rubber plantations. Climate change, on the other hand, may have the opposite effect. Modelling of climate change impacts on rubber production in Yunnan [52] has shown that by 2050 the extremely hot/moist and the extremely hot/mesic bioclimatic zones with optimum conditions for rubber production may have expanded to cover approximately 75% of Xishuangbanna.

## Conclusions

Based on our spatial analyses of rubber expansion we conclude that the expansion of rubber plantations proceeds at an extremely rapid pace that has accelerated in the studied time period from 1988 to 2010, with a clear trend for rubber plantations to expand into higher altitudes. A similar shift towards less optimal sites is inherent in the establishment of more and more plantations on steeper slopes, where growing and working conditions are less favorable and results in an overall decline in mean productivity and economic value per hectare. While planting rubber has up until now improved local livelihoods and contributed to economic growth, these benefits are likely to decrease due to declining prices and expansion into marginally-productive areas. There is also a viable threat to conservation due to the expansion of rubber plantations into protected areas which needs to be tackled urgently by the local government.

The continuing preponderance of smallholder over state plantations and the development of cold-resistant varieties make it possible that the expansion into higher elevations will continue, though it is difficult at this stage to assess the effects of future temperature changes and price fluctuations on this process. Because these high elevation areas harbor most of the remaining forests of Xishuangbanna, declining incomes from newly established plantations will come along with detrimental impacts on biodiversity and ecosystem functions, with broader negative implications for farmers’ livelihoods. It is, therefore, essential that local governments develop long-term land use strategies for sustainable and environmentally-friendly ways of cultivating rubber that balance economic benefits and environmental sustainability. Such strategies must be accompanied by guidance provided to farmers as to which areas are suitable for the growth of rubber and by effective measures of control and law enforcement.

## References

[1] Fox J, Vogler JB. Land-use and land-cover change in montane mainland southeast Asia. Environ Manage. 2005;36(3):394–403. 16132447 10.1007/s00267-003-0288-7

[2] Mann CC. Addicted to Rubber. Science. 2009;325(5940):564–6. 10.1126/science.325_564 19644107

[3] Ziegler AD, Fox JM, Xu JC. The Rubber Juggernaut. Science. 2009;324(5930):1024–5. 10.1126/science.1173833 19460994

[4] Food and Agriculture Organization of the United Nations. FAO Statistical Yearbook 2010. Available: http://www.fao.org/economic/ess/ess-publications/ess-yearbook/ess-yearbook2010/en/. Accessed 16 July 2014.

[5] Li Z, Fox JM. Mapping rubber tree growth in mainland Southeast Asia using time-series MODIS 250 m NDVI and statistical data. Appl Geogr. 2012;32(2):420–32.

[6] Association of Natural Rubber Producing Countries. Nature rubber trends and statistics 2010. Available: http://www.anrpc.org/html/archive.aspx. Accessed 11 December 2014.

[7] National Bureau of Statistics of China. China Statistical Yearbook 2011. Available: http://www.stats.gov.cn/tjsj/ndsj/2011/indexch.htm. Accessed 31 January 2015.

[8] Statistical Bureau of Yunnan Province. Yunnan Statistical Yearbook 2011. Available: http://tongji.cnki.net/kns55/brief/result.aspx?stab=shuzhi&t=1&f=0&tt=%E6%A9%A1%E8%83%B6&areaname=%E8%A5%BF%E5%8F%8C%E7%89%88%E7%BA%B3%E5%82%A3%E6%97%8F%E8%87%AA%E6%B2%BB%E5%B7%9E. Accessed 21 September 2014.

[9] Fox J. The production of forests: tree cover transitions in northern Thailand, northern Laos, and southern China In: Susanna B. Hecht KDM, Christine Padoch, editor. The social lives of forests: Past, present and future of woodland resurgence. Chicago: The University of Chicago Press; 2014 p. 249–59.

[10] Wang F. Thinking of the promotion of private natural rubber industry in Xishuangbanna (In Chinese with English abstract). Trop Agric Sci Technol. 2008;31(2):15–6.

[11] Ministry of Environmental Protection of China. 2011–2030 China Biodiversity Conservation Strategy and Action Plan. Available: http://www.zhb.gov.cn/gkml/hbb/bwj/201009/t20100921_194841.htm. Accessed 31 January 2015.

[12] Li H, Aide TM, Ma Y, Liu W, Cao M. Demand for rubber is causing the loss of high diversity rain forest in SW China. Biod Conserv. 2007;16(6):1731–45.

[13] Li H, Ma Y, Aide TM, Liu W. Past, present and future land-use in Xishuangbanna, China and the implications for carbon dynamics. Forest Ecol Manage. 2008;255(1):16–24.

[14] Li H, Ma Y, Liu W, Liu W. Clearance and fragmentation of tropical rain forest in Xishuangbanna, SW, China. Biod Conserv. 2009;18(13):3421–40.

[15] Liu W, Hu H, Ma Y, Li H. Environmental and socioeconomic impacts of increasing rubber plantations in Menglun Township, southwest China. Mt Res Dev. 2006;26(3):245–53.

[16] Wu ZL, Liu HM, Liu LY. Rubber cultivation and sustainable development in Xishuangbanna, China. Int J Sus Dev World Ecol. 2001;8(4):337–45.

[17] Li Z, Ma Y, Li H, Peng M, Liu W. Relation of land use and cover change to topography in Xishuangbanna, Southwest China. J Plant Ecol. 2008;32(5):1091–103.

[18] Liu X, Feng Z, Jiang L, Li P, Liao C, Yang Y, et al Rubber plantation and its relationship with topographical factors in the border region of China, Laos and Myanmar. J Geogr Sci. 2013;23(6):1019–40.

[19] Ahrends A, Hollingsworth PM, Ziegler AD, Fox JM, Chen H, et al Current trends of rubber plantation expansion may threaten biodiversity and livelihoods. Global Environ Change. 2015; 34: 48–58.

[20] Yi Z-F, Cannon CH, Chen J, Ye C-X, Swetnam RD. Developing indicators of economic value and biodiversity loss for rubber plantations in Xishuangbanna, southwest China: A case study from Menglun township. Ecol Indic. 2014;36:788–97.

[21] Xu JC, Fox J, Vogler JB, Zhang PF, Fu YS, Yang LX, et al Land-use and land-cover change and farmer vulnerability in Xishuangbanna prefecture in southwestern China. Environ Manage. 2005;36(3):404–13. 15995894 10.1007/s00267-003-0289-6

[22] Yu Y. The situation of rubber plantation in upland in Xishuangbanna. Trop Agric Sci Technol (In Chinese with English abstract). 2006;29(03):42–3.

[23] Xishuangbanna Bureau of Statistics. A statistical report on socioeconomic development of Xishuangbanna 2008. Available: http://wenku.baidu.com/link?url=SPdMgKBTeTx8TYOLutwGMILpBO7MOkHqWylTgpCW5rY-erDBm77ziqzTqv7U2CPAfq0zfa6wN55mbTz9KCsxQYR7-PR9nT7UkVZdKknL5HO. Accessed 13 September 2014.

[24] Li Z, Fox JM. Rubber tree distribution mapping in Northeast Thailand. Int J Geosci. 2011;2:573–84.

[25] Dong JW, Xiao XM, Chen BQ, Torbick N, Jin C, Zhang GL, et al Mapping deciduous rubber plantations through integration of PALSAR and multi-temporal Landsat imagery. Remote Sens Environ. 2013;134:392–402.

[26] Li Z, Fox JM. Integrating Mahalanobis typicalities with a neural network for rubber distribution mapping. Remote Sens Lett. 2011;2(2):157–66.

[27] Hurni K. Detection of actual and assessment of potential plantations in Lao PDR using GIS and remote sensing technologies. Bern: University of Bern 2008.

[28] Suratman MN, Bull GQ, Leckie DG, Lemay VM, Marshall PL, Mispan MR. Prediction models for estimating the area, volume, and age of rubber (Hevea brasiliensis) plantations in Malaysia using Landsat TM data. Int Forest Rev. 2004;6(1):1–12.

[29] Zhai D-L, Cannon CH, Slik JWF, Zhang C-P, Dai Z-C. Rubber and pulp plantations represent a double threat to Hainan's natural tropical forests. J Environ Manage. 2012;96(1):64–73. 10.1016/j.jenvman.2011.10.011 22208399

[30] Senf C, Pflugmacher D, van der Linden S, Hostert P. Mapping rubber plantations and natural forests in Xishuangbanna (southwest China) using multi-spectral phenological metrics from MODIS time series. Remote Sens. 2013;5(6):2795–812.

[31] China Meteorological Data Sharing Service System. Available: http://cdc.cma.gov.cn. Accessed 2 October 2014.

[32] Zhang JH, Cao M. Tropical forest vegetation of Xishuangbanna SW China and its secondary changes, with special reference to some problems in local nature conservation. Biol Conserv. 1995;73(3):229–38.

[33] Xu J. The political, social, and ecological transformation of a landscape—The case of rubber in Xishuangbanna, China. Mt Res Dev. 2006;26(3):254–62.

[34] Xu J, Grumbine RE, Beckschaefer P. Landscape transformation through the use of ecological and socioeconomic indicators in Xishuangbanna, Southwest China, Mekong Region. Ecol Indic. 2014;36:749–56.

[35] Su YC, Chong HP. The Utilization of Google Earth Images as Reference Data for The Multitemporal Land Cover Classification with MODIS Data of North Korea. Korean J Remote Sens. 2007;23(5):483–91.

[36] Olofsson P, Foody GM, Herold M, Stehman SV, Woodcock CE, Wulder MA. Good practices for estimating area and assessing accuracy of land change. Remote Sens Environ. 2014; 148: 42–57.

[37] Yi Z. Natural forests and rubber plantations in Xishuangbanna: can market-based ecological compensation mechanisms tip the balance towards restoration? Kunming: Chinese Academay of Sciences; 2012.

[38] Zhuang J, Liang Z, Lin T, De Guzman F. Theory and practice in the choice of social discount rate for cost-benefit analysis: a survey. 2007. ADB. Manila. Available: http://www.adb.org/Documents/ERD/Working_Papers/WP094.pdf. Accessed 10 November 2014.

[39] R Development Core Team. R: A language and environment for statistical computing. Vienna, Austria: R Foundation for Statistical Computing 2013. Available: http://www.R-project.org/. Accessed 10 November 2014.

[40] Association of Natural Rubber Producing Countries. Nature rubber trends and statistics. 2010. Available: http://www.anrpc.org/html/archive.aspx. Accessed 11 December 2014.

[41] Xiao G, Zhong S. Mountain climate with high elevation and rubber suitable region in Xishuangbanna (In Chinese with English abstract). Trop Agric Sci Technol. 2007;30(2):1–6.

[42] Rao PS, Saraswathyamma CK, Sethuraj MR. Studies on the relationship between yield and meteorological parameters of para rubber tree (Hevea brasiliensis). Agric Forest Meteorol. 1998;90(3):235–45.

[43] Priyadarshan PM, Hoa TTT, Huasun H, de Goncalves PS. Yielding potential of rubber (Hevea brasiliensis) in sub-optimal environments. J Crop Improvement. 2005;14(1–2):221–47.

[44] Hu H, Liu W, Cao M. Impact of land use and land cover changes on ecosystem services in Menglun, Xishuangbanna, Southwest China. Environ Monit Assess. 2008;146(1–3):147–56. 18157650 10.1007/s10661-007-0067-7

[45] Zhu H, Xu ZF, Wang H, Li BG. Tropical rain forest fragmentation and its ecological and species diversity changes in southern Yunnan. Biod Conserv. 2004;13(7):1355–72.

[46] Chen L, Dong H, Peng H. Diversity and distribution of higher plants in Yunnan, China. Biod Sci. 2013;21(3):359–63.

[47] Liu J-J, Slik JWF. Forest fragment spatial distribution matters for tropical tree conservation. Biol Conserv. 2014; 171:99–106.

[48] Laurance WF. Averting biodiversity collapse in tropical forest protected areas. Nature. 2012;489(7415):290–4. 10.1038/nature11318 22832582

[49] J. Hammond, Z. Yi, T. McLellan and J. Zhao. Situational Analysis Report: Xishuangbanna Autonomous Dai Prefecture, Yunnan Province, China. ICRAF, Kunming, China, CGIAR Research Program on Integrated Systems for the Humid Tropics. Working paper. 2014; Available: http://english.xtbg.cas.cn/vtxtbg/in/201506/P020150602346580944789.pdf. Accessed 11 January 2016.

[50] Shi, W. 2008. Rubber Boom in Luang Namtha: a Transnational Perspective. Vientiane: GIZ. Available: https://www.mendeley.com/catalog/rubber-boom-luang-namtha-trannational-perspective/. Accessed 27 December 2015.

[51] Shi, W. 2015. Rubber Boom in Luang Namtha: Seven Years Later. Unpublished field notes. Available: http://laofab.org/document/view/2608. Accessed 27 December 2015.

[52] Zomer RJ, Trabucco A, Wang M, Lang R, Chen H, Metzger MJ, et al Environmental stratification to model climate change impacts on biodiversity and rubber production in Xishuangbanna, Yunnan, China. Biol Conserv. 2014;170:264–73.

